# La/Fe-Bimetallic-Modified Red Brick Powder for Phosphate Removal from Wastewater: Characterization, Adsorption, and Mechanism

**DOI:** 10.3390/ma18061326

**Published:** 2025-03-17

**Authors:** Yunrui Zhao, Hui Luo, Rubin Han, Shiheng Tao, Meng Liu, Ming Tang, Jiayao Xing, Limin Chen, Bao-Jie He

**Affiliations:** 1 School of Civil and Ocean Engineering, Jiangsu Ocean University, Lianyungang 222005, China; zhaoyr997997@163.com (Y.Z.); 19816221775@163.com (R.H.); t082701027@163.com (S.T.); lm061615@126.com (M.L.); 18014279115@163.com (M.T.); 2022120879@jou.edu.cn (J.X.); chenliminjou@126.com (L.C.); 2 Institute of Oceanology, Chinese Academy of Sciences, Qingdao 266071, China; 3 Entre for Climate–Resilient and Low–Carbon Cities, Key Laboratory of New Technology for Construction of Cities in Mountain Area, School of Architecture and Urban Planning, Ministry of Education, Chongqing University, Chongqing 400045, China; 4 School of Architecture, Design and Planning, The University of Queensland, Brisbane 4072, Australia

**Keywords:** construction waste, La/Fe modification, domestic sewage, phosphorus removal, magnetism

## Abstract

The use of construction waste red brick powder (RBP) to prepare adsorbents for phosphate removal from wastewater represents a promising technology with substantial research potential. This study investigates the preparation of La-based magnetic red brick powder (La-Fe-RBP) via bimetallic modification to enhance its adsorption performance. The key characteristics, adsorption process, adsorption mechanism, and practical applications of the modified adsorbent were analyzed. The obtained results suggested that the underlying adsorption mechanism of La-Fe-RBP was best described by the Langmuir and pseudo-second-order kinetic models, which suggested that the adsorption mechanism was monolayer chemical adsorption. La-Fe-RBP exhibited rapid kinetics, achieving adsorption saturation in just 40 min, significantly faster than RBP (360 min). Additionally, isotherm experiments determined the highest theoretical adsorption capacity as 42.835 mg/g. More importantly, La-Fe-RBP exhibited efficient phosphate adsorption within a pH ranging from 3 to 8. Furthermore, La-Fe-RBP exhibited high selectivity for phosphate ions in the presence of coexisting ions (${\mathrm{SO}}_{4}^{2-}$, ${\mathrm{NO}}_{3}^{-}$, Cl^−^, ${\mathrm{HCO}}_{3}^{-}$, Mg^2+^, and Ca^2+^), demonstrating its robustness and effectiveness in complex water conditions. FTIR and XPS analyses demonstrated that ligand exchange and electrostatic attraction were the primary mechanisms underlying phosphate adsorption by La-Fe-RBP. Domestic sewage treated with La-Fe-RBP met the Class IV surface water environmental quality standards in China. The findings of this study prove that the La-Fe-RBP composite material, characterized by high adsorption efficiency and strong selectivity, holds significant potential for removing phosphates from real wastewater.

## 1. Introduction

Red bricks are commonly used as a building material in masonry structures due to their porous nature and heat-insulating properties. However, owing to the rapidly growing construction industry in China over the last 50 years, at least 20 billion clay bricks have been produced, many of which have ultimately become construction waste [1]. A large amount of construction waste is sent to the outskirts for open-air piling or landfilling without any treatment, leading to severe environmental pollution [2]. The accumulation of construction waste, when exposed to infiltration, soaking, and rainwater runoff, can generate leachate containing pollutants such as calcium silicate, calcium hydroxide, and sulfate ions. If left uncontrolled, the water resources surrounding construction waste landfill sites may become contaminated, potentially leading to groundwater pollution. This can adversely affect the growth of surrounding plants and animals, jeopardize potable water sources, and lead to significant ecological damage [3].

Numerous researchers and experts have investigated the resource utilization of construction waste, with the goal of reducing environmental pollution through the recycling and reuse of building materials. This approach can contribute to minimizing waste generation and promote the sustainable growth of the construction industry. To tackle the problem of discarded bricks, several solutions have been investigated, such as substituting cement with red brick powder to produce foamed concrete [4], using brick powder to produce a washable water-based coating [5], creating combined curing agents with cement for the stabilization of river dredging sediments [6], and utilizing recycled construction waste bricks as roadbed filler for highways [7]. Additionally, recent studies on sponge cities and constructed wetlands have emerged as a research hotspot, where red brick particles are utilized as filler materials in constructed wetlands to treat pollutants in domestic wastewater [2]. However, the surface of red brick particles primarily consists of inorganic silicates, which restricts their capacity to adsorb organic substances [8]. Therefore, research on modifying red brick powder to create adsorbents and improve adsorption performance has emerged as a promising new direction.

As is well known, phosphorus is essential for plant growth and biological metabolism. However, excessive phosphorus input leads to eutrophication of freshwater systems, posing a threat to the health of humans, animals, and plants. This has emerged as one of the most serious environmental issues, particularly in developing countries [9]. Therefore, controlling external phosphorus inputs has become an urgent necessity to preserve the health of water bodies and ecological balance. The main sources of external phosphorus include agricultural activities, urban domestic wastewater, and industrial effluents. Among these, reducing the external phosphorus load in municipal drainage has become one of the key strategies. To address this issue in municipal drainage, most regions in China have implemented restrictions to ensure that the total phosphorus concentration in discharged wastewater remains below 0.5 mg/L [10]. Consequently, many municipal wastewater treatment plants have adopted various phosphorus control measures to meet these discharge standards. Currently, several methods for phosphorus removal are available, including biological treatment, adsorption, chemical precipitation, and ion exchange. Among these, the adsorption approach is employed extensively to remove phosphate from water owing to its advantages such as a low initial cost, ease of operation, high removal performance, and minimal sludge production. Red brick powder has been shown to be an excellent adsorbent for the removal of phosphate ions due to its graded porous structure, low cost, and good chemical and mechanical stability, as well as its ability to provide active sites for metal ions during modification. The process of modifying red brick powder can enhance its adsorption performance by increasing the number of active sites. Several methods for treating red brick powder for phosphorus removal have been explored, including direct adsorption [11] and a combination of acid modification and calcination [3].

Although the methods mentioned above have improved the ability of red brick powder to remove phosphate to some extent, they are difficult to recycle. Phosphorus is a nonrenewable resource, and recovering it from various waste materials and wastewater is crucial for sustainable development and environmental protection. Therefore, using adsorbents with recyclable and regenerable properties has become an environmentally friendly approach [12]. In this case, if the adsorbent is loaded with iron (Fe), it can be recovered using magnetic force, thereby avoiding secondary pollution. Although previous studies have modified red brick using FeCl_3_ and Al(NO_3_)_3_ to enhance its adsorption capacity (*Q*) [3], a drawback is that these adsorbents do not possess magnetic properties. However, magnetic properties can be imparted to the adsorbent through the method discussed in this study. Even so, a single iron-based adsorbent still has certain drawbacks, such as the tendency to release phosphate in an oxygen-deprived environment [9]. Magnetic lanthanum (La)-based adsorbents have recently attracted considerable attention. La is an eco-friendly and abundant rare earth metal. When added to materials, they can improve their dispersion and serve as active sites, effectively enhancing the material’s ability to adsorb phosphate [13]. Most importantly, La has a strong affinity for phosphate, much higher compared to that of commonly used iron-based and aluminum-based adsorbents, and it rarely releases phosphate again. Furthermore, iron-based adsorbents can better load La onto the surface of the adsorbent through electrostatic interactions [14]. Therefore, compared to magnetic La-based adsorbents, pure Fe-based or pure La-based adsorbents have a higher risk of phosphate release from their surfaces due to changes in the external environment [15]. However, to the best of our knowledge, there is currently limited research on the design and preparation of magnetic La-based construction waste red brick powder adsorbents.

In summary, this study aims to explore the feasibility of using construction waste red brick powder (RBP), a low-cost solid waste material, as a novel adsorbent for removing phosphate ions from water after being co-loaded with La and Fe (La-Fe-RBP). This new adsorbent is expected to have advantages such as environmental friendliness, strong interference resistance, excellent adsorption performance, and recyclability. The main goals of the current study are as follows: (1) synthesize La-Fe-RBP for the efficient separation of phosphate from water; (2) obtain a comparison between the adsorption efficiency of RBP and La-Fe-RBP through adsorption isotherm parameters and adsorption kinetics; (3) explore the impact of the pH and coexisting ions on the adsorption efficiency of the prepared adsorbent; (4) analyze the adsorption mechanism of the synthesized adsorbent; and (5) examine the regeneration performance of the adsorbent and its ability to remove phosphate from real-life sewage. This study not only proposes a unique approach for recycling and utilizing construction waste red bricks but also offers new insights into mitigating water eutrophication, with profound application prospects.

## 2. Materials and Methods

### 2.1. Materials and Chemical Precursors

The chemical precursors utilized in this work were all of analytical grade, including KH_2_PO_4_, LaCl_3_·6H_2_O, HCl, NaOH, FeCl_3_·6H_2_O, FeSO_4_·7H_2_O, NH_4_Cl, NaCO_3_, NaCl, Na_2_SO_4_, and MgCl_2_, all procured from Shanghai National Pharmaceutical Group Chemical Reagents Co., Ltd., Shanghai, China. All solutions in this study were prepared using deionized water.

The red bricks employed in this work were obtained from demolition construction waste at Jiangsu Ocean University (Lianyungang, China). The discarded red bricks were collected, cleaned, and then crushed into particles with a particle size ranging from 0.5 to 5 mm using a jaw crusher (PEX-150 × 250, Henan Fote Machinery Co., Ltd., Zhengzhou, China). The crushed particles were then ground for 2 h in a ball mill (MQGg1212, Henan Fote Machinery Co., Ltd., Zhengzhou, China) to obtain red brick powder. The powder was subsequently cleaned with distilled water, placed inside an oven to dry, and then manually ground and sieved through a 100 μm mesh screen. The final product was red brick powder, referred to as RBP.

### 2.2. Synthesis of La-Fe-RBP

**Figure 1:**
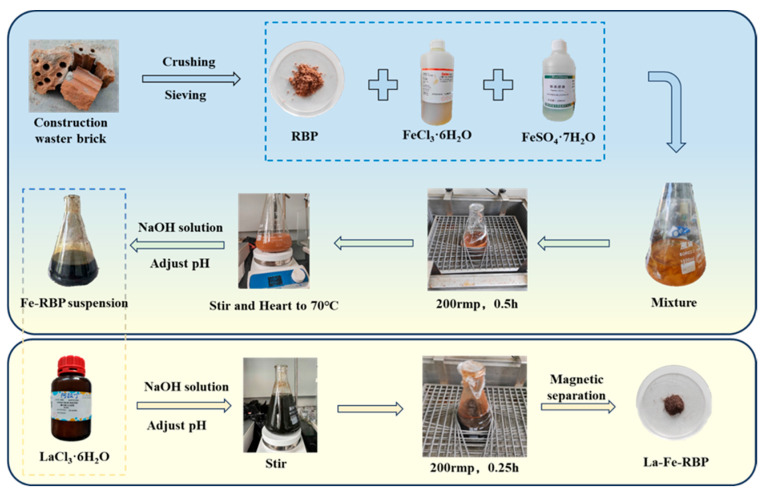
Preparative diagram for La-Fe-RBP.

La-Fe-RBP was synthesized by improving the co-precipitation method based on previous studies [9]. The synthesis process is shown in Figure 1. Briefly, 10 g of RBP was first weighed out and placed in a triangular flask containing 0.25 L of FeCl_3_·6H_2_O (0.1 mol/L) solution and 0.25 L of FeSO_4_·7H_2_O (0.2 mol/L) solution. The mixture was then transferred to a water bath shaker and stirred for 30 min (25 °C). After shaking, the suspension was transferred to a constant-temperature magnetic stirrer and heated at 70 °C. The pH was brought to 10 by adding NaOH, and the resulting solution was further stirred. Finally, the product was collected from the suspension via magnetic separation, washed with deionized water, dried, and ground to obtain Fe-RBP. Next, a suspension containing Fe-RBP was prepared, and 5 g of LaCl_3_·6H_2_O crystals were added to the suspension. The mixture was stirred, and the pH value was brought to 10 by introducing NaOH. The obtained suspension was then placed in the shaker and stirred for another 15 min. The La-Fe-RBP product was collected using the same method as described above.

### 2.3. Material Properties 

The surface morphology of the samples was examined utilizing an FEI QUANTA 250G environmental scanning electron microscopy (SEM) system (FEI Company, Hillsboro, OR, USA), whereas the chemical distributions of the samples before and after modification were determined via its attached energy-dispersive X-ray spectrometer (EDS). The relevant Brunauer–Emmett–Teller (BET) parameters of the samples were measured through an automatic Tristar II 3020 surface area and pore size analyzer (Micromeritics Instrument Corporation, Norcross, GA, USA). The magnetic behavior of the prepared samples was studied using an NBVSM-1 vibrating sample magnetometer (VSM) from Nanbei Instruments (Nanbei Instruments, Zhengzhou, China). The planar structure of the obtained samples was examined via a JEOL JDX-3530X-ray diffraction (XRD) (JEOL Ltd., Tokyo, Japan). A zeta potential analyzer (Litesize DLS, Anton Paar, Graz, Austria) was employed to measure the surface charge of the samples. The surface functional groups of the synthesized specimens were examined via a Nicolet iS5 Fourier-transform infrared spectroscopy (FT-IR) system (Thermo Fisher Scientific, Waltham, MA, USA). The binding energy of the elements within the synthesized samples was obtained using X-ray photoelectron spectroscopy (JEOL JPS-9200, JEOL Ltd., Tokyo, Japan).

### 2.4. Adsorption Test

All batch tests were performed within 250 mL Erlenmeyer flasks at 25 °C and 150 rpm. A specific quantity of the synthesized adsorbent was added to the flask containing a phosphate solution of a specific concentration. The pH of the experimental solution in this study was chosen to be 6.5 in order to better demonstrate its ability to treat actual domestic wastewater. The pH of the resultant mixture was regulated by adding different concentrations of NaOH and HCl solutions (0.1–1 mol/L), and the flask was placed in a shaker for 24 h. After the reaction, the solution was filtered through a 0.45 μm membrane to collect the supernatant, and the residual phosphate concentration was determined using the ascorbic acid-based molybdenum blue method. All experiments were conducted in triplicate, and the mean values of the results along with their associated error bars were reported. The values of the removal rate (*R*, %) and the adsorption equilibrium capacity (*Q*_e_, mg/g) were determined via the formulas provided below:

*R* = [(*C*_0_ − *C*_e_)/*C*_0_] × 100% (1)

*Q*_e_ = [(*C*_0_ − *C*_e_)/m] × *V* (2)

where *C*_e_ (mg/L) and *C*_0_ (mg/L) represent the equilibrium and the initial phosphate concentrations, respectively, whereas *V* (L) and *m* (g) denote the volume of the phosphate solution and the quantity of the adsorbent, respectively.

An adsorbent (75 mg) was mixed with 50 mL of phosphate solution (20 mg/L), and samples were taken at fixed intervals (from 10 to 1440 min) to measure the phosphate concentration value within the obtained mixture. The adsorption process of the phosphate ions by the adsorbent was analyzed based on the pseudo-first-order (PFO) and pseudo-second-order (PSO) kinetic models using the mathematical expressions below:

ln(*Q*_e_ − *Q*_t_) = ln*Q*_e_ − *K*_1_t (3)

*t*/*Q*_t_ = 1/*K*_2_*Q*_e_ + *t*/*Q*_e_ (4)

where *Q*_t_ (mg/g) indicates the adsorption amount at time *t* (min), while *K*_1_ (1/min) and *K*_2_ (g/(mg·min)) indicate the rate constants for the PFO and PSO models, respectively.

The adsorbent (75 mg) was added to phosphate solutions of various concentration values (10–120 mg/L) for the adsorption isotherm tests, with other conditions remaining the same, as described previously. The adsorption behavior of the prepared adsorbent for phosphate was studied based on the Freundlich (Equation (5)), Langmuir (Equation (6)), Temkin (Equation (7)) and Dubinin–Radushkevich (Equation (8)) models:

(5) $$Q_{e}=K_{F}C_{e}^{1/n}$$

*Q*_e_ = *Q*_m_*K*_L_*C*_e_/(1 + *K*_L_*C*_e_) (6)

*Q*_e_ = *BlnA + BlnC*_e_ (7)

*Q*_e_ = *Q*_m_*exp*(−*K*_DR_ ε^2^) (8)

*ε* = *RT ln(1 + 1/C*_e_*)* (9)

*E*^2^ = 1/*2K*_DR_ (10)

In this context, *Q*_m_ (mg/g) denotes the highest value of *Q*; *K*_F_ and 1/*n* are the constants for the Freundlich model; *K*_L_ denotes the Langmuir constant; A and B are the equilibrium binding constant and Temkin equation coefficient, respectively, which is related to heat absorption; *K*_DR_ (mol^2^/kJ^2^) is the constant in the Dubinin–Radushkevich isotherm model; R (J/mol K) and T (K) are the gas constant and absolute temperature, respectively; and E is the adsorption free energy (kJ/mol).

The impact of varying the pH values (2–11) on the phosphate removal efficacy of the synthesized adsorbent (50 mg/L) was investigated. This project aims to study the anti-interference performance of the adsorbent in a complex ionic environment, using common ions (${\mathrm{SO}}_{4}^{2-}$, ${\mathrm{HCO}}_{3}^{-}$, Cl^−^, ${\mathrm{NO}}_{3}^{-}$, Ca^2+^, and Mg^2+^) as interfering ions. The concentration values of the interfering ions were set to 10, 50, and 100 mg/L, with the initial concentration value of the phosphate solution being 20 mg/L.

### 2.5. Recycling Capacity

The recyclability of the adsorbent was evaluated through five cycles of testing. In every cycle, 75 mg of adsorbent was mixed in 50 mg/L of 50 mg phosphate solution. After each adsorption, the adsorbent was desorbed using NaOH solution, followed by cleaning with deionized water. The adsorbent was then dried at a temperature equal to 105 °C and ground for reuse in subsequent cycles.

### 2.6. Remove Phosphorus from Real Sewage

This project aims to use domestic wastewater from the dormitory area of Jiangsu Ocean University as the research object. The components of the wastewater are shown in Table 1. By combining this with indoor simulation experiments, this study systematically investigated the removal efficiency of phosphorus-containing pollutants in urban domestic wastewater using an adsorbent and evaluates its removal performance. A total of 150 mg of adsorbent was added to a container with 100 mL of domestic wastewater. The reaction was carried out on a shaker, and after completion, the obtained mixture was filtered via a 0.45 μm filter membrane for subsequent water quality analysis. The determination of the total phosphorus (TP) in the wastewater was conducted according to the GB 11893-89 Water quality–Determination of total phosphorus–Ammonium molybdate spectrophotometric method [16]. The COD concentrations before and after treatment were measured using a COD meter (NANCHANG HANDR INSTRUMENT CO., LTD, Nanchang, China). The TN and NH_4_^+^-N concentrations in the solution were determined using Nessler’s reagent spectrophotometric method [17], with measurements taken using a reliable UV–vis spectrophotometer (model UV-759S, Jinghua, China).

## 3. Results and Discussion

### 3.1. Material Characterization

**Figure 2:**
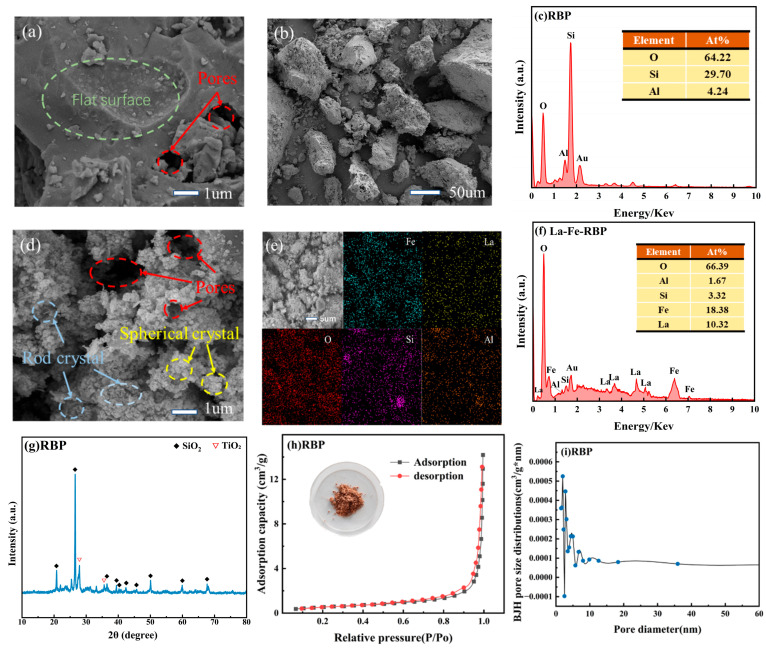

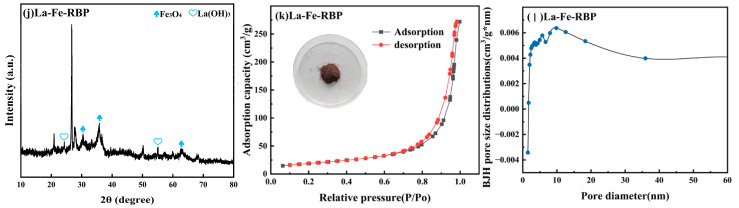
SEM images, EDS data, XRD patterns, N2 adsorption–desorption isotherms, and BJH PSDs of RBP (**a**–**c**,**g**–**i**) and La-Fe-RBP (**d**–**f**,**j**–**l**).

The materials were characterized utilizing the EDS, SEM, BET, and XRD techniques. As depicted in Figure 2a,b, the RBP, after grinding, displays uneven particles, with a flat, large structure present on the surface of individual particles, providing a substrate for the subsequent deposition of metal oxides and hydroxides. The EDS test results of RBP (Figure 2c) indicate that SiO_2_ is the primary component of RBP, which is consistent with previous research findings [14]. In comparison to RBP, Figure 2d displays the SEM images of La-Fe-RBP, which show a significantly rougher surface with a large number of nanoparticles and distinct porous features after modification. This indicates that the La/Fe modification has improved the porous structure of RBP, enhanced the specific surface area, and altered the pore volume, thus providing a higher number of active sites and demonstrating stronger adsorption capability. A huge number of crystalline structures appeared on the surface of La-Fe-RBP, which is believed to be associated with the loading of La/Fe [18]. The elemental distribution mapping image of La-Fe-RBP (Figure 2e) clearly shows the loading of Fe and La, with a significant amount of both elements uniformly distributed on the surface of RBP, indicating successful loading of La and Fe. The EDS spectrum of La-Fe-RBP (Figure 2f) also detected La and Fe, further verifying that the oxides of La and Fe had been loaded onto the adsorbent surface, consistent with the SEM analysis. The total contents of La and Fe in La-Fe-RBP are 10.32% and 18.38%, respectively, which are in close agreement with those reported in previous studies [9].

To investigate the compositional and crystalline changes of RBP and La-Fe-RBP before and after modification, XRD analysis was conducted, with the results presented in Figure 2g,j. The strong peaks detected at 20.88°, 26.7°, 36.44°, 42.38°, 50.08°, 59.94°, and 67.7° correspond to quartz [14], indicating that the main component of RBP is SiO_2_, which is consistent with the EDS analysis results. Compared to RBP, after La/Fe modification, the original SiO_2_ peaks are significantly weakened, while prominent characteristic peaks appear at 30.1°, 35.7°, and 61.2°, corresponding to the characteristic peaks of Fe_3_O_4_ [18]. This indicated that the magnetic Fe_3_O_4_ had successfully been loaded onto the surface of the red brick powder. Notably, distinct peaks at 24.3°and 54.9°, which aligned with the typical signals of La(OH)_3_ [12], suggested that the La on the surface of La-Fe-RBP exists in the form of La(OH)_3_.

The pore size distribution and the N_2_ desorption/adsorption experimental results obtained for RBP and La-Fe-RBP are shown in Figure 2h,i,k,l. According to the IUPAC classification, RBP displayed a type IV isotherm with an adsorption hysteresis phenomenon observed in the relative pressure range of P/P_0_ from 0.6 to 0.9. The hysteresis loop was type H3, which indicated that the material has a mesoporous structure. The pore size of RBP was primarily concentrated within the range from 2 to 50 nm, further supporting its mesoporous nature. For La-Fe-RBP, the nitrogen adsorption–desorption isotherm also showed a type IV characteristic, but lacked a distinct saturation adsorption plateau. The H_3_-type hysteresis loop began at P/P_0_ > 0.6, typically associated with capillary condensation effects in mesoporous materials, further confirming that La-Fe-RBP is indeed mesoporous [14]. The pore size distribution, as shown in Figure 2l, indicates that La-Fe-RBP’s pore sizes are also concentrated in the 2 to 50 nm range, reflecting its mesoporous structure, which enhances its adsorption capabilities. The BET surface area calculations for RBP and La-Fe-RBP are presented in Table 2. After La/Fe modification, the surface area of La-Fe-RBP significantly increased to 67.59 m^2^/g, compared to 1.855 m^2^/g for RBP, indicating that the La-Fe modification greatly enhances the surface area, providing more adsorption sites for phosphates. Additionally, La-Fe-RBP shows a pore volume equal to 0.399 cm^3^/g and a mean pore diameter equaling 22.687 nm, both of which are considerably higher than RBP’s values of 0.019 cm^3^/g and 8.855 nm. This increase likely results from the surface loading and agglomeration of Fe/La on RBP. The porous structure facilitates the effective transfer of phosphates from the surface to the interior pores, significantly increasing the adsorption rate and allowing for rapid attainment of adsorption equilibrium [10].

### 3.2. Adsorption Kinetics

**Figure 3:**
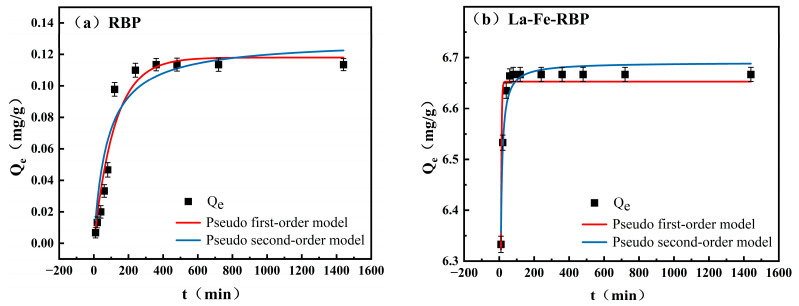
Adsorption kinetic curves: (**a**) RBP; and (**b**) La-Fe-RBP. (The pH of the solution in the kinetics experiment is 6.5).

The adsorption equilibrium time can significantly influence the performance, economic viability, and sustainability of adsorption systems, making it one of the important considerations in designing and optimizing such systems. The kinetic behavior of RBP and La-Fe-RBP composites was studied by increasing the contact time from 0 to 1440 min, as illustrated in Figure 3. It can be observed from Figure 3a that RBP’s adsorption of phosphates was primarily concentrated in the first 120 min, reaching 86.3% of the adsorption limit (0.0978 mg/g). After that, the amount of P adsorbed increased slowly, reaching saturation around 360 min, with a final adsorption limit of only 0.1135 mg/g. In comparison, the La-Fe-RBP exhibited a significantly higher value of *Q* for low-concentration phosphates, as shown in Figure 3b. La-Fe-RBP rapidly adsorbed within the first 10 min, achieving 94% of its adsorption limit (6.333 mg/g). Subsequently, as the adsorption sites on the La-Fe-RBP surface became progressively occupied, phosphates entered the interior of the composite, leading to a reduction in the rate of adsorption. About 60 min later, it reached saturation at 6.667 mg/g. Thus, it is evident that after modification with La/Fe, RBP significantly shortens the adsorption time for P, achieving rapid kinetics.

Both the PFO and the PSO kinetic models were utilized to fit the adsorption data and acquire a deeper understanding of the adsorption control mechanism during the adsorption process. The fitted data are provided in Table 3. Although the fitting formulas are different, the adsorption capacities are similar. The theoretical adsorption capacities of RBP and La-Fe-RBP based on the PFO (0.118 mg/g, 6.652 mg/g) and the PSO kinetic models (0.129 mg/g and 6.690 mg/g) were quite close to the experimentally obtained values (0.1135 mg/g and 6.667 mg/g). The R^2^ values for the PFO kinetic model are 0.8823 for La-Fe-RBP, while the R^2^ values for the PSO kinetic model are 0.9584 for La-Fe-RBP. These findings showed that the PSO kinetic model provided a better description of the adsorption process, suggesting that the adsorption process of La-Fe-RBP is primarily a chemical adsorption [12].

### 3.3. Adsorption Isotherm

**Figure 4:**
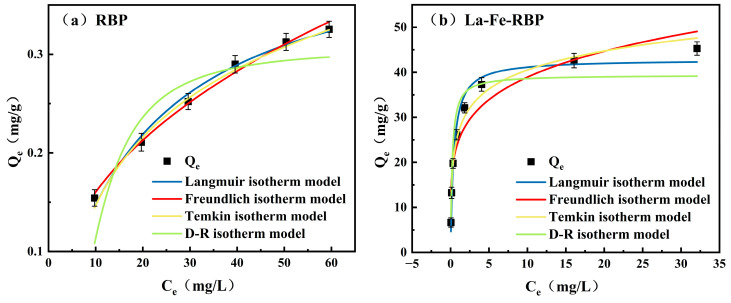
Adsorption isotherm curves: (**a**) RBP; and (**b**) La-Fe-RBP. (The pH of the solution in the isotherm experiment is 6.5).

The adsorption isotherm is essential for evaluating the performance of adsorbents. Figure 4 illustrates the adsorption behavior of RBP and La-Fe-RBP at varying initial phosphate concentration values. For RBP, the value of *Q* decreased as the initial phosphate concentration increased, reaching a maximum of 0.4264 mg/g. In contrast, the *Q* value of La-Fe-RBP increases steadily with phosphate concentration and eventually reaches saturation at higher concentrations. The high concentration of phosphate solution provided the driving force for mass transfer during the initial adsorption phase, accelerating the movement of phosphate ions from the solution to the adsorbent surface [12].

The extracted experimental data were fitted based on the Freundlich and Langmuir models to understand the internal adsorption mechanism better, and the corresponding parameter values are provided in Table 4. For RBP, the fit of the Freundlich model (R^2^ = 0.991) was better compared to that of the Langmuir model (R^2^ = 0.988), indicating that the adsorption process involves multilayer adsorption, which is consistent with other research findings [19]. In contrast, for La-Fe-RBP’s adsorption of phosphate, the Langmuir model provides a better fit (R^2^ = 0.966) in comparison to the Freundlich model (R^2^ = 0.896), suggesting that the adsorption process occurred uniformly on the surface of the complex, forming a monolayer adsorption phenomenon [18].

The theoretical maximum *Q* value of La-Fe-RBP was found to be approximately 42.835 mg/g based on the Langmuir model, which was substantially higher compared to the *Q* value obtained for RBP (0.4264 mg/g). This substantial increase in *Q* value following La/Fe modification enhances the effectiveness of the modified RBP as a phosphate adsorbent. Furthermore, the *Q* value of La/Fe-modified materials was compared with other La-based adsorbents, as given in Table 5. The obtained results demonstrated that the bimetallic modification exhibited a higher value of *Q* in comparison to the monometallic modification. This is due not only to the increased number of active sites provided by bimetallic modification, but also to the fact that the addition of Fe optimizes the electronic structure of La, thereby significantly enhancing the adsorption ability [20]. In addition, the magnitude of E in the D-R model is commonly employed to estimate the adsorption reaction mechanism. As demonstrated in Table 4, the E values obtained from Equation (10) range from 8 to 16 kJ/mol, suggesting that the process is a chemical adsorption process [21]. These results are consistent with the previous kinetic model analysis.

### 3.4. Impact of pH and Coexisting Ions

Previous works have demonstrated that pH is an important factor affecting the adsorption efficiency of adsorbents. It directly influences the ionic form of phosphorus and its chemical behavior in solution, in addition to the self-stability and surface charge of the adsorbent [25]. Additionally, lanthanum modification was performed under alkaline conditions by adding NaOH (Equation (11)). We conducted a point of zero charge (PZC) test, and the data indicated that the PZC of La-Fe-RBP is 6.46. When the pH is lower than 6.46, the surface of the adsorbent carries a positive charge. This is due to the high proton concentration in the acidic environment, which causes the hydroxide ions (OH^−^) in La-OH to easily combine with protons, forming the positively charged La-${OH}_{2}^{+}$. (Equation (12)) [12].

**Figure 5:**
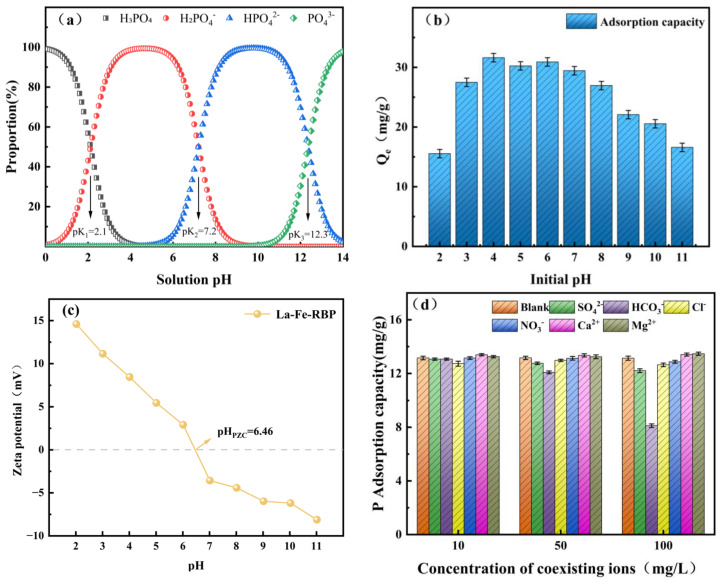
Species distribution of phosphate examining different pH levels (**a**); the effect of initial pH (**b**); the zeta potential analysis (**c**); and the effect of coexisting ions on La-Fe-RBP (**d**) [31].

As shown in Figure 5a, the forms of phosphate vary at varying pH values. When pH < 2.1, H_3_PO_4_ is the predominant species, and metal oxides are readily protonated in acidic environments, acquiring a positive charge. As a result, there is negligible electrostatic interaction or ion exchange between phosphate and La-Fe-RBP, resulting in extremely weak adsorption [22]. Between pH 2.1 and 7.20, H_2_PO_4−_ is the dominant species, and under acidic conditions, the lanthanum-based surface acquires a positive charge. Electrostatic attraction (Equations (13) and (14)) and ion exchange (Equation (15)) between the positive charge on the La-Fe-RBP surface and the oxyanions in the phosphate can enhance the value of *Q* [26]. Between pH 7.20 and 12.3, HPO_4_^2−^ is the predominant species. At this point, the pH has already exceeded the PZC. Metal oxides are more readily deprotonated, and the surface primarily acquires a negative charge. This results in apparent electrostatic repulsion (Equation (16)) between phosphate and the La-Fe-RBP surface. Additionally, the higher adsorption free energy of HPO_4_^2−^ restricts the ligand exchange between La-OH and HPO_4_^2−^, weakening the counteracting effect and further reducing the value of *Q* [19]. These results indicate that electrostatic attraction is an essential mechanism for the adsorption of PO_4_^3−^ ions by La-Fe-RBP.

LaCl_3_ + 3NaOH → La(OH)_3_ + 3NaCl (11)

(12) $$\mathrm{La-OH}+H^{+}↔\mathrm{La}-{\mathrm{OH}}_{2}^{+}$$

(13) $${\mathrm{La-OH}}_{2}^{+}+H_{2}{\mathrm{PO}}_{4}^{-}\to (\mathrm{La}-{\mathrm{OH}}_{2}^{+})\;(H_{2}{\mathrm{PO}}_{4}^{-})$$

(14) $${\mathrm{La-OH}}_{2}^{+}+{\mathrm{HPO}}_{4}^{2-}\to (\mathrm{La}-{\mathrm{OH}}_{2}^{+})\;({\mathrm{HPO}}_{4}^{2-})$$

(15) $${\mathrm{La-OH}}_{2}^{+}+H_{2}{\mathrm{PO}}_{4}^{-}\to \mathrm{La}-H_{2}{\mathrm{PO}}_{4}^{-}+H_{2}O$$

(16) $$\mathrm{La}\equiv \mathrm{OH}+{\mathrm{HPO}}_{4}^{2-}\to {\left({\mathrm{LaHPO}}_{4}\right)}^{+}+3{\mathrm{OH}}^{-}$$

Due to the complexity of wastewater, efficient adsorbents must be capable of resisting interference from coexisting ions in practical applications. Therefore, this study investigates the selectivity of La-Fe-RBP for phosphate adsorption, examining the effects of several representative competitive ions (${\mathrm{SO}}_{4}^{2-}$, ${\mathrm{NO}}_{3}^{-}$, Cl^−^, ${\mathrm{HCO}}_{3}^{-}$, Ca^2+^, and Mg^2+^), as shown in Figure 5c. At low concentrations, these coexisting ions do not significantly impact phosphate adsorption, with phosphate removal rates by La-Fe-RBP exceeding 94% in their presence. This indicates that at low concentrations, these competitive ions are unable to compete with phosphates ions for adsorption sites on the surface of the La-Fe-RBP sample. When their concentration reaches 100 mM, the influence of ${SO}_{4}^{2-}$, ${NO}_{3}^{-}$, and Cl^−^ is minimal. Phosphate is classified as a hard base, and thus hard acids should be chosen, which is consistent with the fact that metals such as Fe^3+^, Al^3+^, Mg^2+^, and Ca^2+^ tend to form stable complexes with phosphate. Although Cl^−^, and ${SO}_{4}^{2-}$ are also considered hard bases, their Lewis basicities differ, with the order being ${PO}_{4}^{2-}$ > ${SO}_{4}^{2-}$> Cl^−^ > ${NO}_{3}^{-}$. This makes phosphate a stronger electron pair donor, and thus more likely to form a coordination complex with metal ions such as La [27]. However, the impact of _${HCO}_{3}^{-}$_ on the *Q* value of La-Fe-RBP is more significant and negative compared to the other ions. This phenomenon is attributed to several factors. First, excessive hydrolysis of _${HCO}_{3}^{-}$_ inevitably increases the solution’s pH [28]. As the pH rises with increasing ion concentration, the distribution of phosphate ions changes, which in turn affects the *Q* value. Second, unlike the other three anions, _${HCO}_{3}^{-}$_ has a similar ionic structure to phosphate. It tends to react with La ions on the La-Fe-RBP surface, resulting in intense competition with phosphate in solution and forming La_2_(CO_3_)_3_ precipitates. This significantly reduces the adsorbent’s ability to absorb phosphate [29]. In addition, the addition of Ca^2+^ and Mg^2+^ not only did not inhibit phosphate adsorption but actually enhanced the adsorption efficiency. This phenomenon may be attributed to the ability of Ca^2+^ and Mg^2+^ to precipitate with phosphate, which simultaneously reduces the electrostatic repulsion between phosphate and La-Fe-RBP, providing additional adsorption sites for phosphate [30].

### 3.5. FTIR and XPS

**Figure 6:**
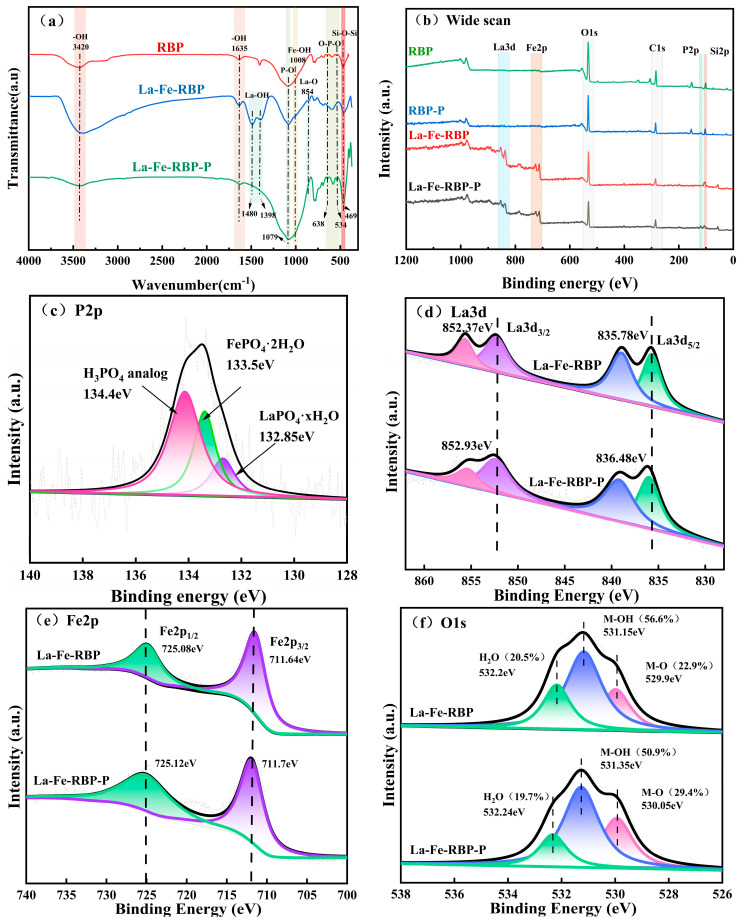
FTIR spectra of RBP and La-Fe-RBP (**a**); XPS spectra of RBP and La-Fe-RBP (**b**); (**c**) p 2p; (**d**) La 3d; (**e**) Fe 2p; and (**f**) O 1s.

Figure 6a presents the FTIR profiles obtained for the RBP, La-Fe-RBP, and La-Fe-RBP-P samples. Both adsorbents displayed bending vibration and asymmetric stretching peaks of the Si-O-Si bond at approximately 1079 cm^−1^ and 874 cm^−1^, which are characteristic peaks of red brick powder. These findings are consistent with the previous XRD results.

The infrared bands found at 3406 cm^−1^ and 1635 cm^−1^ within the spectrum were attributed to the stretching vibration of the -OH groups from physically adsorbed water on the material. The enhancement of these peaks following modification was ascribed to the loading of metal hydroxides and oxides [14]. In the La-Fe-RBP-P spectrum, the peaks at these positions diminish and vanish, suggesting that the -OH groups underwent ligand exchange with phosphate during the adsorption process [24]. The vibration at 1008 cm^−1^ is likely attributed to Fe-OH hydroxyl groups [32], suggesting that iron has been loaded onto the surface of RBP. Following the adsorption process, the disappearance of the Fe-OH peak indicated an interaction between phosphate and iron, resulting in the development of Fe-P complexes [15]. A small peak around 854 cm^−1^ may be attributed to La-O [2]. New peaks at 1480 cm^−1^ and 1398 cm^−1^ in the La-Fe-RBP composite spectrum are attributed to La-OH bond vibrations, confirming the successful loading of lanthanum as lanthanum hydroxide on the La-Fe-RBP surface [23]. After phosphorylation, these peaks disappeared, and new signals at 534 and 638 cm^−1^ appeared, corresponding to O-P-O bending vibrations, indicating an intra-layer complexation reaction between P and La during the adsorption process. Apart from the bending vibration peaks of O-P-O at 534 and 638 cm^−1^, a signal appearing at 1079 cm^−1^ was ascribed to the asymmetric P-O stretching vibrations, which coincided with the asymmetric Si-O-Si stretching vibration, leading to a broader and more intense peak after adsorption [23].

Additionally, XPS characterization was performed prior to and following the adsorption process to investigate the underlying adsorption mechanism of La-Fe-RBP for phosphates further. Figure 6b presents the full-scale spectra of RBP and La-Fe-RBP, showing the elements before and after adsorption. It is clearly observed that after modification, the signals of La and Fe are detected, indicating the successful loading of the bimetals onto the surface of RBP. After adsorption, P signals are observed in both the raw and modified spectra, with the peak intensity being more prominent after modification, confirming that the prepared La-Fe-RBP successfully and efficiently captured phosphates.

The high-resolution spectrum of the P element presented in Figure 6c revealed the existence of the P 2p peak occurring at 133.5 eV. Compared to the standard P 2p peak (134.0 eV) within the KH_2_PO_4_ spectrum, this peak was at a lower energy value, which indicated the formation of a new phosphate complex [33]. Such peak shifts are commonly observed in phosphate adsorption onto metal-based materials, suggesting that La-Fe-RBP has a significant affinity for phosphates and a strong interaction between them [15]. The proximity of this peak to the reference samples FePO_4_·2H_2_O (133.6 eV) and LaPO_4_·2H_2_O (132.8 eV) further confirmed the generation of inner-layer Fe-P and La-P complexes. Figure 6d shows the La 3d spectrum, which presents a characteristic double-peak pattern. Before adsorption, the binding energy values for La 3d_3/2_ and La 3d_5/2_ were 852.37 eV and 835.78 eV, respectively. Following the adsorption process, the binding energies increased by 0.56 eV and 0.7 eV, respectively, which indicated electron transfer in the valence band of La during the adsorption process. This energy shift suggests the formation of a coordination complex and the creation of La-O-P inner-sphere complexes [34]. In contrast to La 3d, the Fe 2p spectrum (Figure 6e) showed minimal changes before and after adsorption, with only slight shifts in the Fe 2p_1/2_ (725.08 eV to 725.4 eV) and Fe 2p_3/2_ (711.64 eV to 711.8 eV) peaks, showing only small shifts of 0.04 eV and 0.06 eV, respectively. This indicated that iron played a minor role in the adsorption of phosphates, with only a small portion of Fe involved in the development of inner-sphere Fe–phosphate complexes [35]. Figure 6f shows the O1s spectrum, where the three peaks are assigned to the different forms of oxygen in the La-Fe-RBP composite. After adsorption, significant changes were observed: the relative area ratio of the La-OH peak reduced from 56.6% to 50.9%, while the relative area ratio of the M-O peak enhanced from 22.9% to 29.4%. This suggests that the phosphate removal occurred through ligand exchange between -OH groups and phosphate ions, which then coordinated with the La and a small number of Fe active sites. This mechanism is the primary pathway for La-Fe-RBP to adsorb phosphates [15].

### 3.6. The Recyclability of La-Fe-RBP

**Figure 7:**
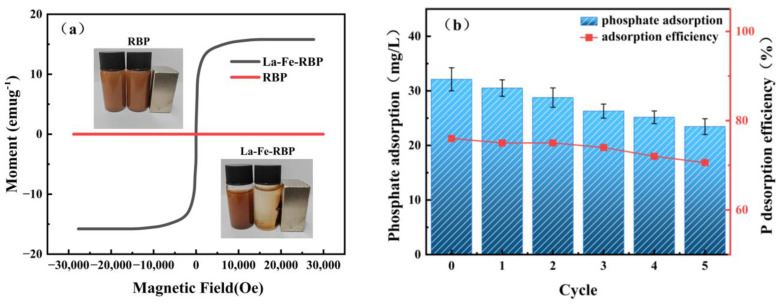
VSM curves (**a**); and recirculation experiments (**b**).

Magnetism is essential for evaluating the recyclability of magnetic adsorbents. Figure 7a illustrates the magnetic properties of RBP and La-Fe-RBP. It is evident that RBP itself does not exhibit magnetic properties. In contrast, La-Fe-RBP displays distinct magnetic characteristics, with a saturation magnetization value equal to 15.79 emu g^−1^. Magnetic strength is crucial for the repeated use of recyclable adsorbents. Prior studies have shown that La-based magnetic adsorbents typically do not exhibit high saturation magnetization owing to the hybridization of La-based compounds with Fe_3_O_4_ particles [36]. Herein, the saturation magnetization of La-Fe-RBP was lower compared to that of pure Fe_3_O_4_ (63.80 emu g^−1^) but higher in comparison to most other previously reported La-modified adsorbents, such as LaCl_3_-modified magnetic zeolite (5.74 emu g^−1^) [37], LaCl_3_-modified magnetic carbon microspheres (1.58 emu g^−1^) [19], and NaLa(CO_3_)_2_-Fe_3_O_4_ hybrid magnetic adsorbents (8.23 emu g^−1^) [38]. This suggests that La-Fe-RBP exhibited better recyclability for the adsorption of the PO_4_^3−^ ions. The recovery performance of both materials under an external magnetic field is illustrated in Figure 7. RBP contains suspended particles and remains unaffected by the magnetic field. In contrast, the clear water surrounding La-Fe-RBP indicates the absence of suspended particles, and the material is quickly attracted by a magnet. This magnetic property of the composite material facilitates easy separation from water, enabling both the recovery of the adsorbent and the extraction and utilization of adsorbed phosphate.

Cyclic adsorption capacity is an important evaluation criterion for magnetic adsorbents. In this study, La-Fe-RBP was used for cyclic desorption tests, and Figure 7b displays the phosphate adsorption capacity of La-Fe-RBP after five adsorption cycles. It can be observed that during the first cycle, La-Fe-RBP still exhibited a value of *Q*, with the adsorption efficiency for phosphate remaining higher than 95%. However, the value of *Q* gradually decreased in subsequent cycles. After completing five cycles, the adsorption efficiency of La-Fe-RBP still reached 73%, and the phosphate desorption rate exceeded 70% in all cases. This suggests that during the desorption process, La-P precipitates deposit on the adsorbent surface, leading to permanent occupation of some adsorption sites. As a result, the active components and surface porosity of the adsorbent decrease, thereby reducing the *Q* value [33]. Nevertheless, the overall adsorption performance remains satisfactory, and the material can be effectively recycled, suggesting that La-Fe-RBP holds promising environmental benefits and application potential.

### 3.7. Real Sewage Treatment

**Figure 8:**
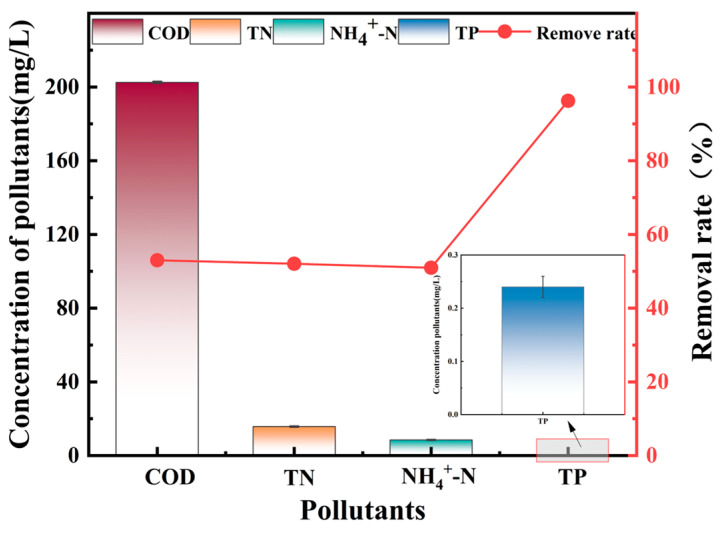
Treated effluent pollutant concentrations.

To address and prevent eutrophication in water bodies and control phosphate levels in municipal wastewater, it is necessary to evaluate the adsorption efficiency of the adsorbents in real wastewater. This study investigates the phosphate removal efficiency of La-Fe-RBP in domestic wastewater to assess its practical application. The wastewater used in the study was collected from a student dormitory, with the water quality characteristics shown in Table 1. Figure 8 illustrates the pollutant concentrations after treatment with La-Fe-RBP, all demonstrating high removal efficiency. The concentrations of COD, TN, and NH_4_^+^-N significantly decreased, with removal rates exceeding 50%. The final concentrations were 202.48 mg/L, 115.718 mg/L, and 8.509 mg/L, respectively. Notably, the total phosphorus concentration reached 0.24 mg/L, which complies with the Chinese Surface Water Quality Standard Class IV (TP = 0.3 mg/L). These results highlight the practical applicability of La-Fe-RBP for removing phosphate ions from real wastewater. However, the removal efficiency of La-Fe-RBP in actual wastewater is lower than that observed in laboratory-prepared phosphate solutions, likely due to the more complex composition of domestic wastewater.

## 4. Conclusions

This study investigates the carrier properties of construction waste red brick powder (RBP) and the strong affinity of Fe and La for phosphates. By using a chemical co-precipitation method, La-Fe-RBP was successfully synthesized.

(1) Due to the incorporation of iron and lanthanum, La-Fe-RBP exhibits significant roughness, porosity, and magnetism. La-Fe-RBP demonstrates superior adsorption kinetics and capacity compared to RBP, reaching saturation in approximately 60 min. The PSO model can better describe the adsorption kinetics of the adsorbent, indicating that the adsorption process is primarily chemical adsorption. Isotherm studies show that the Langmuir model can effectively describe the phosphate loading behavior on La-Fe-RBP, with a maximum theoretical *Q* value of 42.835 mg/g;
(2) Batch adsorption tests showed that La-Fe-RBP maintained a high value of *Q* across a wide range of pH values (3 to 8) and demonstrated high selectivity for phosphate, even in the presence of competing ions, with minimal interference at low concentrations. The results obtained from the XPS and FTIR characterizations proved that the adsorption mechanism involved ligand exchange and electrostatic attraction, forming a metal–O–P inner complex on the surface of La-Fe-RBP.
(3) La-Fe-RBP can be successfully recovered magnetically and retains good phosphate adsorption efficiency even after five desorption/adsorption cycles. In the treatment of real wastewater, it still meets the Class IV surface water environmental quality standards of China.

In summary, the results above indicate that the preparation of La-Fe-RBP serves as an efficient and selective adsorbent. It not only effectively addresses water body nutrient overload but also provides a means for the beneficial use of solid waste red brick powder, contributing to the concepts of energy conservation, emission reduction, and green development. At the same time, improving the desorption efficiency of the adsorbent and preventing the leaching of metal oxides will be key issues for future research. Overall, La-Fe-RBP can be considered an economically viable adsorbent for phosphate removal from wastewater.

## Figures and Tables

**Table 1:** Real domestic sewage quality.

Index	pH	COD (mg/L)	TN (mg/L)	NH_4_^+^-N (mg/L)	TP (mg/L)
Value	6.92 ± 0.2	382 ± 5	30.2 ± 0.2	16.7 ± 0.3	5.9 ± 0.1

**Table 2:** The obtained values of total pore volume, mean pore diameter, and BET specific surface area for the RBP and La-Fe-RBP samples.

Samples	BET Surface Areas (m^2^/g)	Total Pore Volume (cm^3^/g)	Mean Pore Size (nm)
RBP	1.855	0.019	8.855
La-Fe-RBP	67.59	0.399	22.687

**Table 3:** The acquired adsorption kinetics parameter values for the RBP and La-Fe-RBP samples.

Adsorbents	T (°C)	PFO Model			PSO Model		
		K_1_ (1/min)	Q_e_ (mg/g)	R^2^	K_2_ (g/mg·min)	Q_e_ (mg/g)	R^2^
RBP	25	0.0083	0.1180	0.9445	0.0991	0.1292	0.9621
La-Fe-RBP	25	0.3004	6.6527	0.8823	0.2839	6.6907	0.9584

**Table 4:** Parameters of adsorption isotherms of RBP and La-Fe-RBP.

Adsorbents	T (°C)	Langmuir Model			Freundlich Model			Temkin Model			D-R Model		
		**Q_m_**	**K_L_**	**R^2^**	**K** **_F_**	**1/n**	**R^2^**	**A**	**B**	**R^2^**	**K**	**E**	**R^2^**
RBP	25	0.426	0.053	0.988	0.062	0.410	0.991	0.435	0.100	0.991	86.42	0.076	0.911
La-Fe-RBP	25	42.83	2.417	0.966	24.63	0.199	0.896	77.01	6.094	0.987	0.0067	8.639	0.924

**Table 5:** A comparison of the P adsorption characteristics for various La-based adsorbents.

Materials	Absorption Capacity (mg/g)	Experimental Conditions (Temperature, Dosage, P Initial Concentration)	Reference
La-Fe-RBP	42.84	25 °C; 1.5 g/L; 10–120 mg/L	This Work
Activated carbon fiber–La–OH	15.3	25 °C; 2.5 g/L;10–70 mg/L	[22]
Mesoporous silica–La	27.98	25 °C; 0.8 g/L; 5–80 mg/L	[23]
La–zeolite synthesized from fly ash	21.48	25 °C; 10 g/L; 5–300 mg/L	[17]
La–Zr@Fe_3_O_4_	49.1	25 °C; 0.25 g/L; 0–50 mg/L	[24]
Magnetic lanthanum/iron-modified bentonite	14.3	25 °C; 2 g/L; 2–40 mg/L	[9]

## Data Availability

The original contributions presented in this study are included in the article. Further inquiries can be directed to the corresponding authors.
